# Supplementary material for: Flexible High-Performance and Screen-Printed Symmetric Supercapacitor Using Hierarchical Rodlike V3O7 Inks
Source: Nanomaterials (Basel). 2023 Aug 8;13(16):2282. doi: 10.3390/nano13162282 (PMC10457910; doi:10.3390/nano13162282)
Supplement: Supplementary file 1 [file nanomaterials-13-02282-s001.zip › nanomaterials-2540329-supplementary.pdf]

Supporting Information

Flexible high-performance and screen-printed symmetric supercapacitor using hierarchical rodlike V_3O_7 inks

Baoying Lin [1], Yinyin Zheng [1], Jinglu Wang [1], Qian Tu [1], Wentao Tang [1,*], Liangzhe Chen [1,*]

[a] School of Electronic Information Engineering, Jingchu University of Technology, Jingmen 448000, China

*Corresponding authors.

E-mail addresses: chen_lz1991@jcut.edu.cn (L. Chen), twt@whu.edu.cn (W. Tang)

1. Preparation of the $V_3O_7{\cdot}H_2O$ template

The $V_3O_7{\cdot}H_2O$ template was synthesized by a hydrothermal method according to the early work [1]. Generally, 0.72 g V_2O_5 powder was dispersed 60 mL H_2O with ceaseless stirring. Then, 5 mL H_2O_2 (30wt%) was added dropwise and stirred for 1 h. The resulting solution was transferred to a Teflon container and kept at 180°C for 48 h. The $V_3O_7{\cdot}H_2O$ powder was collected after washing with ethanol and H_2O and freeze-drying.

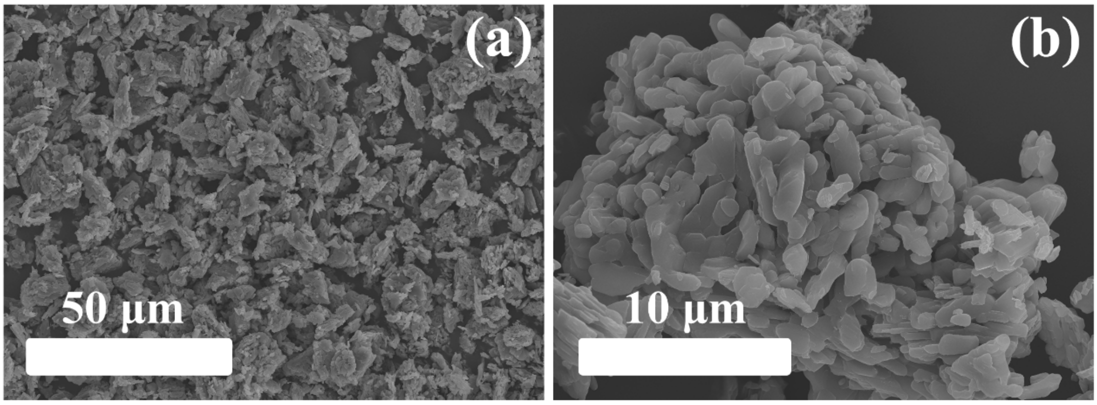

Figure S1. SEM images of the V_2O_5 nanoparticles with (a) low magnification and (b) high magnification.

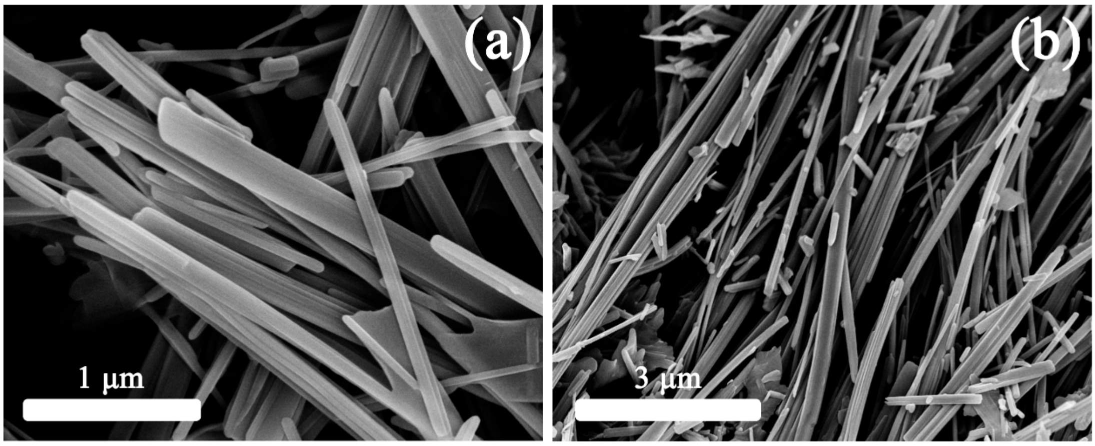

Figure S2. SEM images of the $V_3O_7·H_2O$ template with (a) high magnification and (b) low magnification.

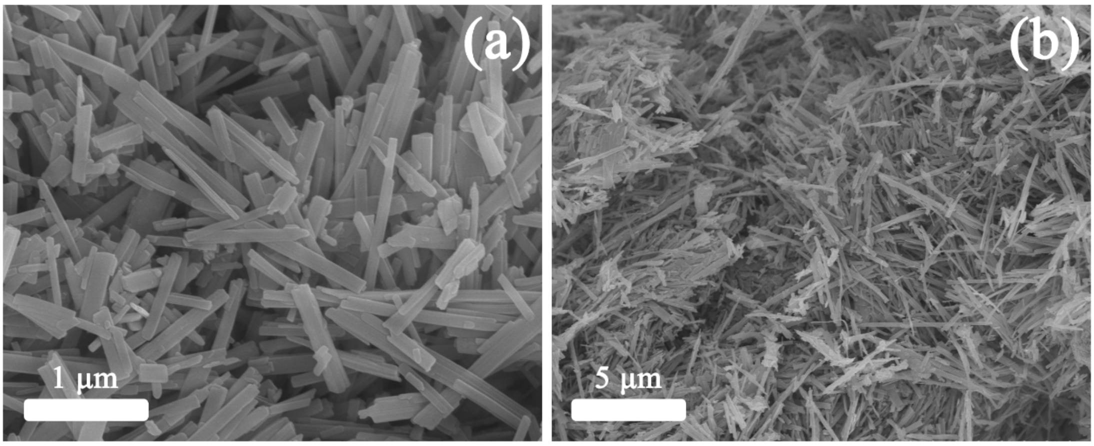

Figure S3. SEM images of the $V_3O_7{\cdot}H_2O$ nanorod with (a) high magnification and (b) low magnification.

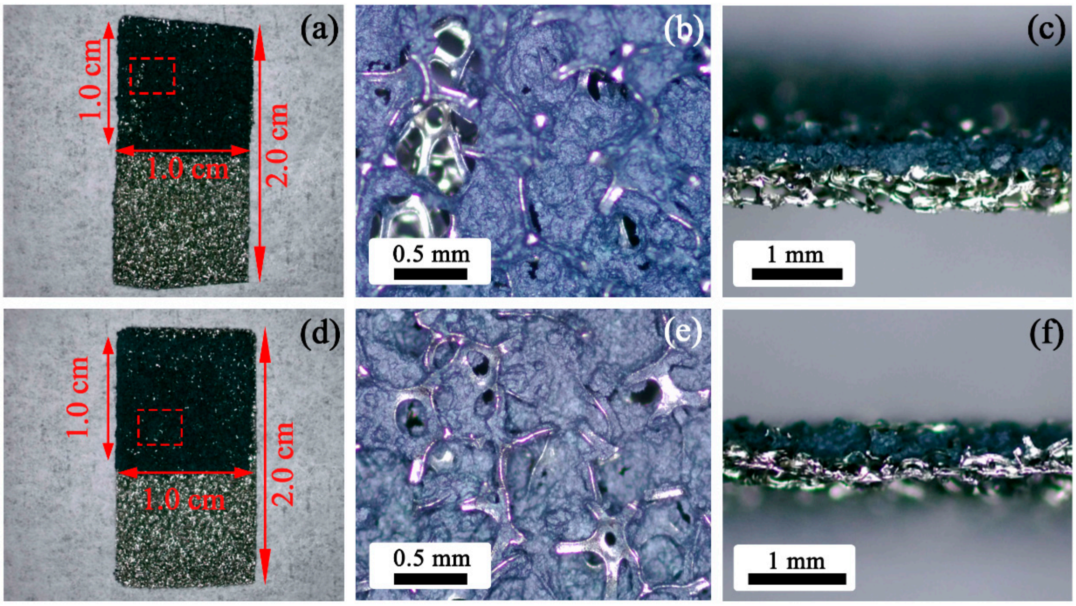

Figure S4. (a) The top view, (b) high-magnification and (c) cross-sectional images of the SP-60 electrode; (d) the top view, (e) high-magnification and (f) cross-sectional images of the SP-100 electrode.

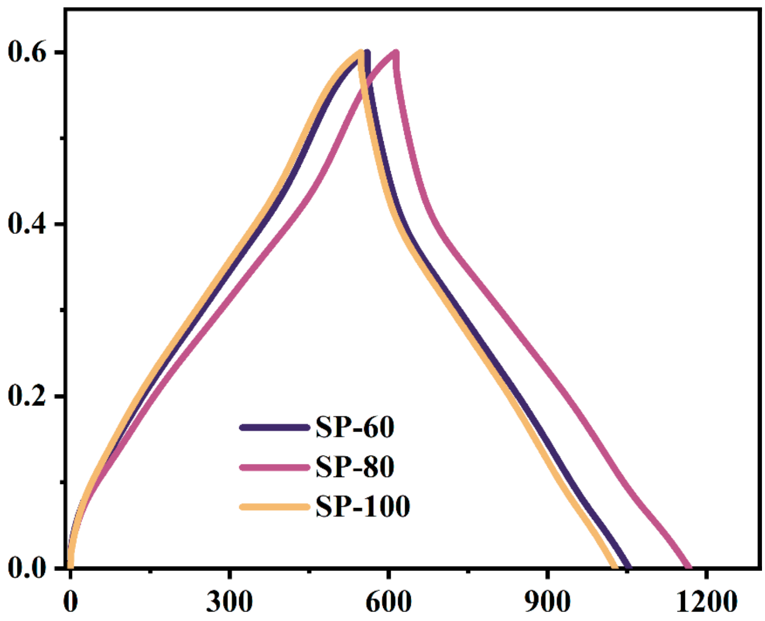

Figure S5. GCD curves at a current density rate of 0.3 A/g of SP-60, SP-80 and SP-100 electrodes.

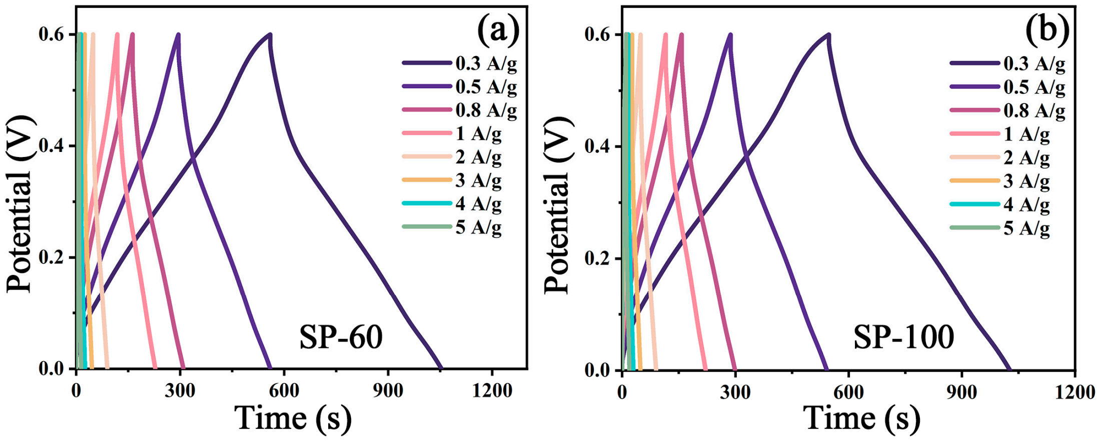

Figure S6. GCD curves of (a) SP-60 and (b) SP-100 electrodes at different current density rates.

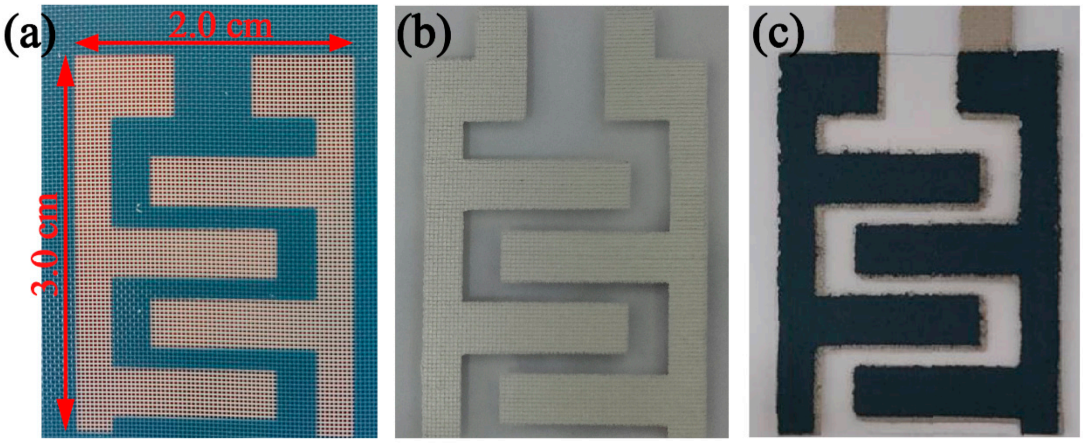

Figure S7. Optical photos of (a) 80-meshes plate with an interfinger pattern, (b) Ag paste interfinger pattern, (c) V_3O_7 supercapacitor without PVA/Na_2SO_4 gel.

Table S1. Impedance values of the SP-60, SP-80 and SP-100 before cycling according to the equivalent circuit.

Electrodes	R_s (Ω)	R_{ct} (Ω)	*CPE* (F)	*W* (Ω)
SP-60	3.31	0.30	1.02	0.14
SP-80	3.01	0.10	1.03	0.07
SP-100	3.11	0.25	0.88	0.05

Table S2. Comparison of areal power density and energy density for V_3O_7 SSC and other reported vanadium oxide-based supercapacitors.

Supercapacitors	Areal energy density	Areal power density	Refs.
V_3O_7 SSC	129.45 μWh/cm^2	0.42 mW/cm^2	this work
α-V_2O_5 SSC	0.48 μWh/cm^2	0.11 mW/cm^2	[2]
V_2O_5/FTO SSC	7.70 μWh/cm^2	0.36 mW/cm^2	[3]
Double-layer VO_2 SSC	0.80 μWh/cm^2	0.02 mW/cm^2	[4]
$V_2O_5 \cdot H_2O$/graphene SSC	1.13 μWh/cm^2	0.01 mW cm^2	[5]
V_2O_5@PEDOT/graphene SSC	0.18 μWh/cm^2	0.01 mW/cm^2	[6]
MXene-TiS_2//MWCNTs-VO_2 ASC	32.50 μWh/cm^2	1.20 mW/cm^2	[7]

References:

[1] Liu, Y.; Zhang, P.; Wang, X.; Sun, J.; Sun, J.; Wen, Z. Fabrication of $V_3O_7 \cdot H_2O$/graphene cathode for high performance zinc-Ion batteries. *Mater. Lett.* **2022**, *317*, 132124.

[2] Adewinbi, S.A.; Busari, R.A.; Animasahun, L.O.; Omotoso, E.; Taleatu, B.A. Effective pseudocapacitive performance of binder free transparent α-V_2O_5 thin film electrode: Electrochemical and some surface probing. *Physica B* **2021**, *621*, 413260.

[3] Azadian, F.; Rastogi, A.C. Energy storage performance of thin film nanocrystalline vanadium oxide with fluorinated tin oxide current carrier electrode for solid-state transparent supercapacitors based on ionic liquid gel electrolyte. *Electrochim. Acta* **2020**, *330*, 135339.

[4] Alhebshi, N.A.; Vaseem, M.; Minyawi, B.A.; AlAmri, A.M.; Shamim, A. Single and double layer of monoclinic VO_2 ink-based printed and interdigitated supercapacitors. *Energy Technol.* **2022**, *10*, 2200432.

[5] Bao, J.; Zhang, X.; Bai, L.; Bai, W.; Zhou, M.; Xie, J.; Guan, M.; Zhou, J.; Xie, Y. All-solid-state flexible thin-film supercapacitors with high electrochemical performance based on a two-dimensional $V_2O_5 \cdot H_2O$/graphene composite. *J. Mater. Chem. A*, **2014**, *2*, 10876-10881.

[6] Wang, L.; Shu, T.; Guo, S.; Lu, Y.; Li, M.; Nzabahimana, J.; Hu, X. Fabricating strongly coupled V_2O_5@PEDOT nanobelts/graphene hybrid films with high areal capacitance and facile transferability for transparent solid-state supercapacitors. *Energy Storage Mater.* **2020**, *27*, 150-158.

[7] Zhao, B.; Wang, S.; Yu, Q.; Wang, Q.; Wang, M.; Ni, T.; Ruan, L.; Zeng, W. A flexible, heat-resistant and self-healable "rock-ing-chair" zinc ion microbattery based on MXene-TiS_2 (de) intercalation anode. *J. Power Sources* **2021**, *504*, 230076.
